# A Multi-Hemagglutinin-Based Enzyme-Linked Immunosorbent Assay to Serologically Detect Influenza A Virus Infection in Animals

**DOI:** 10.3390/vetsci6030064

**Published:** 2019-07-22

**Authors:** Miki Okumura, Akiko Takenaka-Uema, Shin Murakami, Taisuke Horimoto

**Affiliations:** Department of Veterinary Microbiology, Graduate School of Agricultural and Life Sciences, University of Tokyo, 1-1-1 Yayoi, Bunkyo-ku, Tokyo 113-8657, Japan

**Keywords:** influenza virus, hemagglutinin, subtype, enzyme-linked immunosorbent assay, boar, raccoon

## Abstract

Mammals can play a role as an intermediate host in the emergence of mammalian-adapted reassortants or mutants of avian influenza A viruses, with pandemic potential. Therefore, detecting viral infection in animals followed by assessment of the hemagglutinin (HA) subtype of the agent is an indispensable process for risk assessment in pandemic preparedness. In this study, we tested the potential of an enzyme-linked immunosorbent assay as a rapid diagnosis method, using a panel of HA subtype antigens. By analyzing reference immune sera, we found that this novel assay could detect HA subtype-specific antibodies without considerable inter-subtypic cross-reactivities, contributing to diagnosis of influenza virus infection.

## 1. Introduction

The natural reservoirs of influenza A viruses, except subtypes H17 and H18 bat-derived viruses [1,2], are aquatic birds. However, these viruses are occasionally transmitted to fowl or mammalian animals, causing diseases. Although most influenza cases are transient, in rare cases, an influenza A virus can cause a pandemic by jumping species barriers and establishing a new lineage in a new host. To date, the H1, H2, H3, H5, H6, H7, H9, and H10 subtypes of influenza A viruses have been shown to infect humans [3,4,5,6,7]. Among these subtypes, only H1, H2, and H3 viruses have currently or historically become epidemic in humans as seasonal influenzas following pandemics. 

In past pandemics, pigs played a role in the emergence of pandemic viruses as a mixing vessel between avian and human influenza viruses, as both viruses could infect this animal because of the presence of avian-type (α2–3 linked sialic acid) as well as human-type (α2–6 linked sialic acid) receptors on its respiratory epithelial cells [8]. However, some other mammalian animals also possess both types of sialic acid receptors on the cells [9], suggesting that they can act as mixing vessels in which novel viruses with pandemic potential can emerge.

To serologically detect influenza virus infections in animals, the agar gel immunodiffusion test is widely and routinely used as a type-specific assay but reportedly less sensitive in some wild animals [10]. Several commercial kits based on blocking or competitive enzyme-linked immunosorbent assays (ELISAs) are available [11] and useful for screening with a number of test samples from several animals. These assays detect specific antibodies to a viral nucleoprotein (NP) that possesses common antigenicity among influenza A viruses. Since these ELISA kits do not provide information on the hemagglutinin (HA) subtypes of the viruses, hemagglutinin-inhibition (HI) and viral-neutralizing (VN) tests, using each HA subtype virus antigen, are additionally or primarily used to determine the HA subtypes of the viruses [12]. However, these two tests require biosafety level 2 or 3 laboratories, as infectious viruses are used as antigens. In addition, the VN test takes a long time to obtain results. 

In this study, we investigated a multi-HA-based ELISA to serologically and rapidly detect HA subtype-specific antibodies in sera from virus-infected animals and showed its potential as a rapid diagnosis method for detection of influenza virus infections. 

## 2. Materials and Methods

### 2.1. Cells and Viruses

Madin–Darby canine kidney (MDCK) cells, obtained from the American Type Culture Collection (ATCC; CCL-34), were maintained in minimal-essential medium (MEM) supplemented with 5% newborn calf serum (NCS) and antibiotics. Human influenza virus, A/California/04/2009 (H1N1pdm), was propagated in MDCK cells in serum-free MEM with 0.3% bovine serum albumin in the presence of 1 µg/mL of tosylsulfonyl phenylalanyl chloromethyl ketone (TPCK)-trypsin (Worthington, Lakewood, NJ, USA). Other influenza viruses, including A/duck/Hokkaido/17/2001 (H2N3), A/duck/Mongolia/301/2001 (H3N2), A/duck/Hokkaido/1058/2001 (H4N5), A/chicken/Yamaguchi/8/2004 (H5N1), A/duck/Hokkaido/228/2003 (H6N8), A/quail/Aichi/1/2009 (H7N6), A/turkey/Ontario/6118/1968 (H8N4), A/Hong Kong/1073/1999 (H9N2), A/duck/Hokkaido/18/2000 (H10N4), A/duck/England/1/1956 (H11N6), A/duck/Alberta/60/1976 (H12N5), A/duck/Hokkaido/W186/2006 (H13N6), A/mallard/Astrakhan/263/1982 (H14N5), and A/duck/Australia/341/1983 (H15N8), were propagated in 10-day-old embryonated chicken eggs at 37 °C, and the allantoic fluids containing viruses were collected and stored at −80 °C. Viruses were kindly gifted from Dr. Kawaoka (Division of Virology, Institute of Medical Science, University of Tokyo) and Dr. Sakoda (Laboratory of Microbiology, Graduate School of Veterinary Medicine, Hokkaido University).

### 2.2. Antisera

Chicken hyperimmune antisera to A/Narita/1/2009 (H1N1pdm), A/swine/Shimane/221/1999 (H2N3), A/duck/Hokkaido/49/1998 (H9N2), A/chicken/Germany/N/49 (H10N7), A/duck/Alberta/60/1976 (H12N5), or A/duck/Australia/341/1983 (H15N8) were kindly provided by the Laboratory of Microbiology, Graduate School of Veterinary Medicine, Hokkaido University. 

To obtain mouse antisera to subtypes H3, H4, H5, H7, H8, H11, H12, H13, and H14 viruses, each virus described above was concentrated and partially purified through 25% sucrose cushion by ultracentrifugation at 100,000 × *g* for 2 h. A viral pellet was dissolved in phosphate-buffered saline (PBS) and used for immunization to mice. The female 6-week-old mice (ICR; Shizuoka Animal, Shizuoka, Japan) were intracutaneously inoculated with each HA subtype virus mixed with an adjuvant (ADJUVANT 10; GERBU Biotechnik GmbH, Heidelberg, Germany) three times at a 2-week interval followed by bleeding to obtain antisera.

### 2.3. Cloning of HA Genes in Plasmid

The RNA was extracted from the allantoic fluid of chicken eggs containing each HA subtype virus. Reverse transcription of viral RNA was performed using primers containing the conserved sequences at the 3′ ends of the viral segments and SuperScript III reverse transcriptase (Life Technologies Japan, Tokyo, Japan). PCR was conducted using a specific primer pair for each *HA* gene segment and HotStar HiFidelity polymerase (QIAGEN, Tokyo, Japan). Primer sequences will be provided upon request. PCR products were purified using the Fast Gene Gel/PCR Extraction kit (NIPPON Genetics, Tokyo, Japan) and cloned into pCR2.1-TOPO or pCR-BluntII-TOPO (Life Technologies, Carlsbad, CA, USA). *HA* genes in these plasmids were sub-cloned into a pCAGGS vector containing the blasticidin S-resistant gene. Each construct was sequenced using specific primers in an automated sequencer (Life Technologies Japan, Applied Biosystems 3130xl, Tokyo, Japan) to authenticate its sequence.

### 2.4. Expression of HAs in Transfected Cells

Each plasmid was transfected into MDCK cells by using polyethylenimine (PEI; Polysciences, Inc., Warrington, PA, USA), and the transfected cells were cultured in MEM containing 5% fetal bovine serum and blasticidin S (10 μg/mL) and limited-diluted to obtain stable HA-expressing cells. Expression of each HA in the cells was detected in an immunostaining assay using chicken or mouse antisera to HAs described above. To confirm HA expression on the cell surface, we used a fusion assay. Briefly, the cells were treated with TPCK-trypsin (5 μg/mL) for 15 min in the serum-free medium and with citrate phosphate buffer (pH 4.9) for 3 min and cultured in MEM containing 5% NCS for 24 h at 37 °C. Giemsa staining was used to visualize fused cells under a microscopy.

### 2.5. Multi-HA-Based Enzyme-Linked Immunosorbent Assay

To prepare antigens for a multi-HA-based enzyme-linked immunosorbent assay (HA-ELISA), the cells expressing each HA subtype were collected by a cell scraper, washed with PBS, and lysed by addition of 1% Nonidet P-40 in PBS for 15 min at 4 °C. After centrifugation, the cell lysate containing each HA subtype from H1 to H15 was diluted with a 0.015 M carbonate-0.035 M bicarbonate buffer (pH 9.6) into 50 μg/mL and dispensed into duplicate wells (50 μL/well) of an ELISA microplate (Nunc Maxisorp; Thermo Fisher Scientific, Tokyo, Japan). Equivalent concentrations of HA antigens among subtypes were assessed by an immunoblot assay. The normal cell lysate was also prepared as a control antigen under the same procedures. After incubation at 4 °C overnight, the wells were washed with PBS containing 0.05% Tween 20 (PBS-T), and blocking solution (Block Ace; Dainippon Pharmaceutical, Osaka, Japan) (200 μL/well) was added, followed by incubation at 37 °C for 1 h. After washing with PBS-T, duplicate wells were added with test sera (50 μL/well) diluted in PBS-T. A dilution of 1:500, 1:1000, or 1:5000 was used for mouse or chicken hyperimmune antisera for assay validation. In addition, 1:200 dilution of sera from wild animals was used to efficiently detect specific antibodies in test samples. After incubation at 37 °C for 1 h, the wells were washed with PBS-T, and 50 μL of diluted peroxidase-conjugated protein A/G (1:2000; ProZyme, San Leandro, CA, USA) or rabbit antibody to chicken IgG (1:2000; MP Biomedicals/Cappel, Irvine, CA, USA) was added to the wells. After incubation at 37 °C for 1 h, the wells were washed with PBS-T, and substrate solution containing 3,3′,5,5′-tetramethylbenzidine (50 μL/well) (Pierce TMB Substrate Kit; Thermo Fisher, Waltham, MA, USA) was added, followed by incubation for 20 min at 23 °C. The enzyme reaction was terminated by the addition of 1N H_2_SO_4_ (25 μL/well). The optical density (OD) value at 450–650 nm was determined with an ELISA reader (iMark; BioRad, Tokyo, Japan). Serum is defined as positive to an HA subtype-specific antibody if the OD value with this HA subtype antigen, which was estimated by subtraction of the value without test serum from the value with test serum, was more than the mean OD value plus three times the standard deviation (SD) with the other 15 antigens comprising 14 HA subtypes and a control.

### 2.6. Animal Serum Samples

Sera (n = 6) collected from wild boars (*Sus scrofa leucomystax*) in Japan were used to estimate reaction specificity of HA-ELISA; four of them were antibody positive to H1N1 but negative to H3N2 and H5N1 viruses using our previous VN test, while the others were negative to these three subtypes [13]. In addition, three sera collected from apparently healthy feral racoons (*Procyon lotor*) captured in Japan, which were antibody positive to H5N1 (n = 1) [14] or to H9N2 (n = 2) (our unpublished data), respectively, by VN test, were used for multi-HA-ELISA. Prior to the test, all sera were treated with a receptor destroying enzyme (RDEII; Denka Seiken, Tokyo, Japan) for 16 h at 37 °C followed by inactivation for 30 min at 56 °C. The sera were further treated with kaolin and with MDCK cell pack (1 × 10^5^ cells/μL) for 16 h at 4 °C to decrease non-specific reactions. 

### 2.7. Virus-Neutralization Test

We performed a VN test with 100 PFU of A/chicken/Yamaguchi/8/2004 (H5N1) or A/Hong Kong/1073/1999 (H9N2) in MDCK cells according to the WHO manual [12]. VN titer was shown as the reciprocal of the highest serum dilution that completely inhibited virus replication on duplicate wells of 96-well microplates.

### 2.8. Ethics Statement

Our animal study protocol was conducted in accordance with the Regulations for Animal Care at the University of Tokyo and was approved by the Animal Experiment Committee of the Graduate School of Agricultural and Life Sciences at the University of Tokyo.

## 3. Results and Discussion

To prepare antigens for a multi-HA-based ELISA, we first constructed a series of plasmids expressing each HA subtype from H1 to H15 by cloning each HA gene from viruses into a protein-expression vector. By transfection of these plasmids into MDCK cells, we selected stable cell lines expressing each HA subtype. These cells formed syncytia using a fusion assay, authenticating cell surface expression of functional HAs (Figure S1). We prepared lysates of these HA-expressing cells and used them as antigens for multi-HA-ELISA, by which the HA subtype-specific antibody could be distinctively detected. We also utilized a protein A/G conjugate that could bind immunoglobulins from vast animal species for color development of positive reactions, expecting application of this assay to sero-surveillance for wild animals.

**Figure 1:**
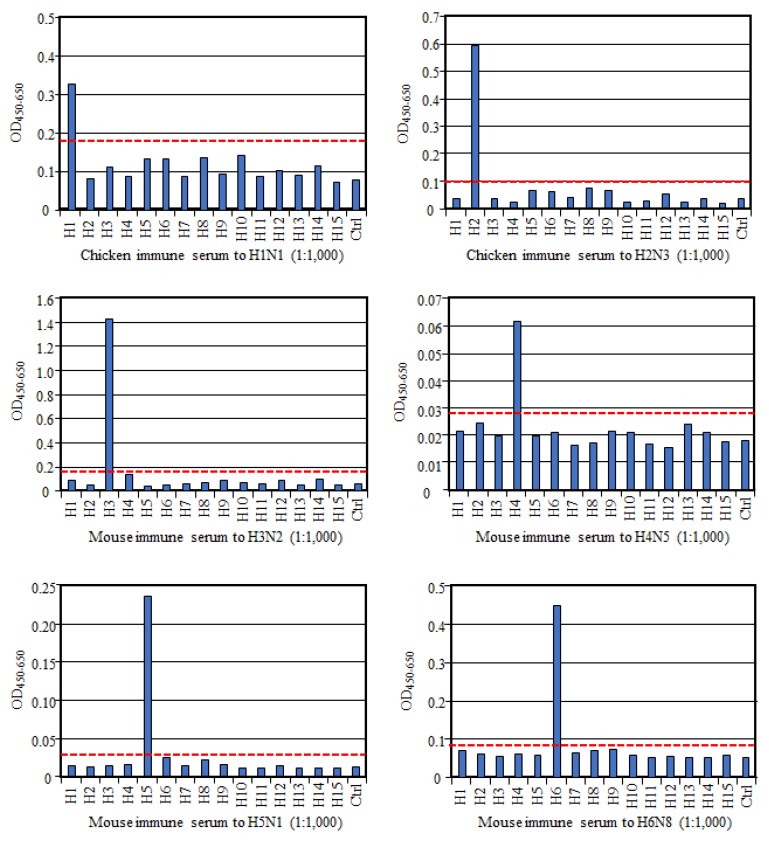

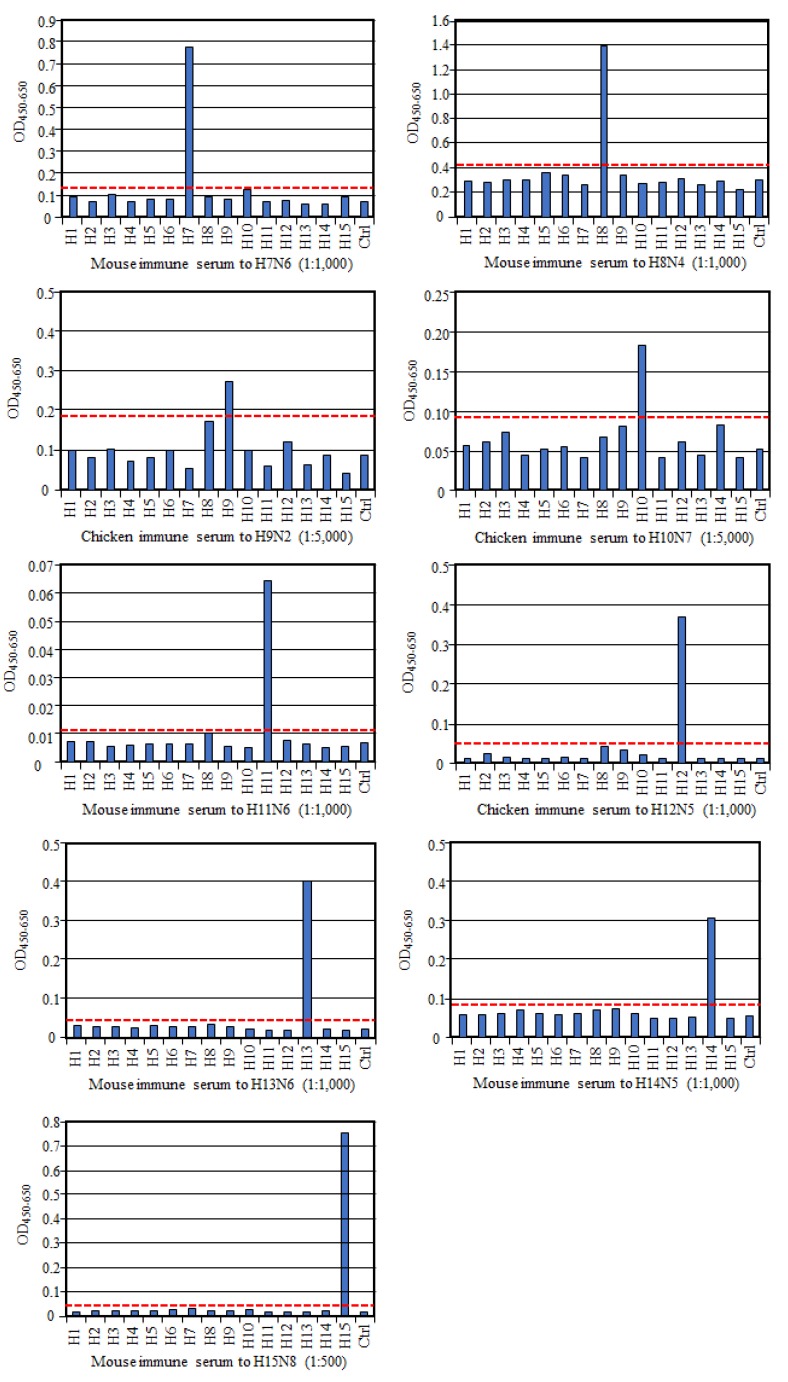
Validation of the multi-hemagglutinin (HA)-ELISA. Specificity of the assay was evaluated using chicken or mouse antisera to each HA subtype. With each antiserum (diluted at 1:500–1:5000), the optical density (OD)_450–650_ values to H1–H15 and control antigens are shown. The red dotted line indicates the cut-off value, which is the mean OD value plus three times the standard deviation (SD) with the other 15 antigens comprising 14 HA subtypes and a control. Representative results from multiple experiments are shown in the figure.

To evaluate if HA-ELISA could detect an HA subtype-specific antibody, we prepared antisera to each HA subtype and examined their reactivities in this assay. As shown in Figure 1, all these reference antisera, except anti-H9 serum, reacted only to the homologous HA subtype, much more than to all the other heterologous HA subtypes and the control antigen, indicating no or very low cross-reactivity between HA subtypes. Exceptionally, antisera to the H9 subtype reacted not only to the homologous H9 antigen but also to the H8 subtype antigen albeit weakly, suggesting cross-reactivity between these two HA subtypes. However, according to the cut-off value we proposed here, the OD value with H8 antigen was judged as a negative result. Collectively, these data authenticate reaction specificity of our HA-ELISA to any HA subtype.

**Figure 2:**
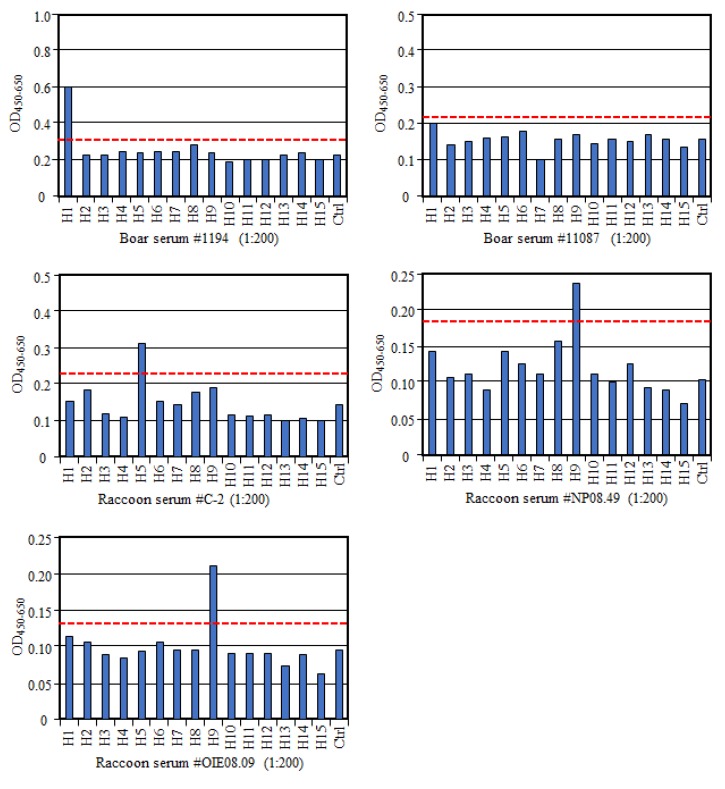
Confirmation of the specificity of the multi-HA-ELISA using wild animal samples. Boar serum #1194 and #11087 were viral neutralizing (VN) positive to H1N1 in the VN test. Raccoon serum #C-251 were VN positive to the H5N1 virus. Raccoon sera #NP08.49 and #OIW08.09 were VN positive to the H9N2 virus. The red dotted line indicates the cut-off value. Representative results from multiple experiments are shown in the figure.

To further confirm the specificity of this multi-HA-ELISA, we tested six sera from wild boars, including four VN antibody positives and two antibody negatives to the H1N1pdm virus. We found that three of VN positive samples with VN titers of 1024 (sample #1194), 1024 (#11123), and 12,800 (#21362) [13] were also positive to H1 antigen in the multi-HA-ELISA, whereas one VN positive sample (#11087; VN titer of 128) was negative in this ELISA (Figure 2). Since this sample showed the lowest VN titer among the VN positive samples, this discrepancy in the result may suggest lower sensitivity of this ELISA compared with the VN test. In contrast, all VN negative sera were negative in the HA-ELISA as well. In addition, we tested the raccoon sera, which possessed VN antibodies to H5N1 (sample #C-2; VN titer of 256) [14] or H9N2 viruses (#NP08.49 and #OIE08.09; VN titers of 8) in multi-HA-ELISA and found that all three samples were positive to H5 or H9 antigen in ELISA as well (Figure 2). Although these results confirm specificity of multi-HA-ELISA, we need to consider some difference in sensitivity between the VN test and ELISA for their usage. Our multi-HA-ELISA used the full-length HAs as antigens, but its specificity and sensitivity might increase by using the globular head domain of HAs as antigens [15], as antibodies binding the stem region of HA might exhibit possible cross-reactivities among subtypes [16].

Although an HI test detects subtype-specific antibodies, nonspecific inhibitors of HA contained in sera of animals may cause false positives if the inhibitors are not removed from the sera. The VN tests are highly specific and sensitive in the detection of subtype-specific antibodies and can also detect strain-specific antibodies. We conclude that the multi-HA-ELISA established herein may be used as an additional assay method for serological surveys of influenza viruses in animals. As a recommendation, the HA-ELISA may be used to identify an HA subtype of the positive samples following the commercial competitive ELISA with an NP antigen, providing rapid and high-throughput testing for influenza virus infections. However, to widen its usage, multiple HA antigens in each HA subtype (e.g., H1 and H3) should be included in the assay and multiple viral infections with different HA subtypes should be specifically detected. In addition, we need to determine how long the HA-coated ELISA plate can be stored, which is important for the practical use of this assay in the future. Although serological methods are not used for early detection of influenza virus infections, they contribute to the retrospective investigation of the epidemiology of outbreaks. Our multi-HA-ELISA could be useful for such studies.

In particular, the multi-HA-ELISA, which does not require host-specific secondary antibodies, has the potential to be widely used for sero-surveillance of wild mammals. Because of their behavior, they can transmit the viruses among wild and domestic animals. Viral transmission and expansion on poultry farms may cause considerable economic losses. In addition, those on pig farms can be a risk factor for the emergence of mutant viruses infecting humans with a pandemic potential. Thus, continuous monitoring of the exposure of wild mammals to avian influenza viruses should be considered. Further investigations with surveillance of influenza virus infections in peridomestic animal species are needed to better understand the influenza ecology, as well as for the risk assessment in pandemic preparedness. 

## Supplementary Materials

The following is available online at https://www.mdpi.com/2306-7381/6/3/64/s1, Figure S1: Syncytium formation by HA expressed in MDCK cells.
