# Supplementary material for: A Multi-Hemagglutinin-Based Enzyme-Linked Immunosorbent Assay to Serologically Detect Influenza A Virus Infection in Animals
Source: Vet Sci. 2019 Jul 22;6(3):64. doi: 10.3390/vetsci6030064 (PMC6789898; doi:10.3390/vetsci6030064)
Supplement: Supplementary file 1 [file vetsci-06-00064-s001.pdf]

Supplementary Information

A multi-hemagglutinin-based enzyme-linked immunosorbent assay to serologically detect influenza A virus infection in animals

Miki Okumura, Akiko Takenaka-Uema, Shin Murakami and Taisuke Horimoto

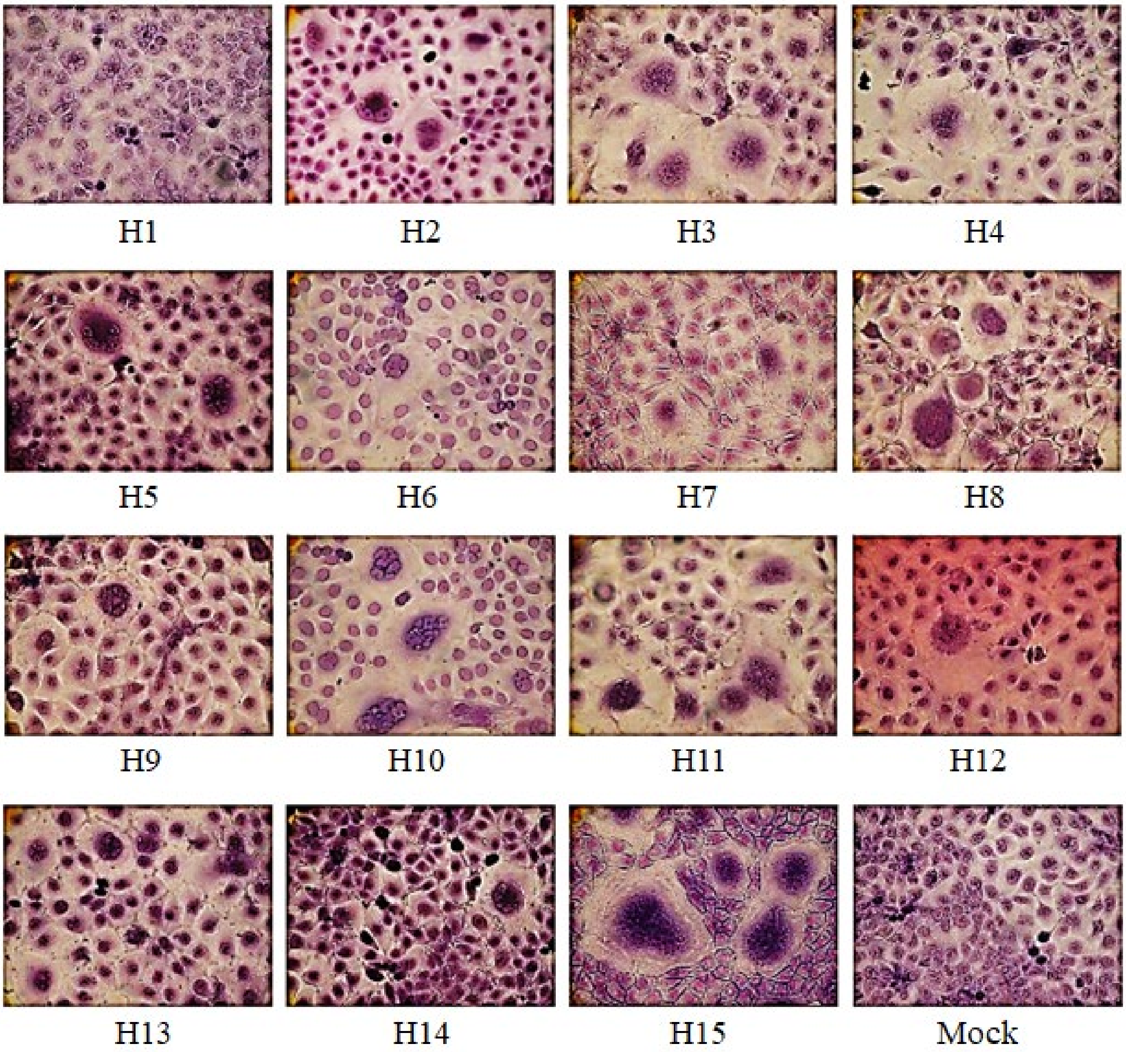

Fig. S1. HA expression in the transfected MDCK cells. Expression of each subtype HA was confirmed by a fusion assay in which syncytium was observed on cell culture via Giemsa staining.
